# Reciprocal regulation of metabolic and signaling pathways

**DOI:** 10.1186/1471-2164-11-197

**Published:** 2010-03-24

**Authors:** Andreas S Barth, Ami Kumordzie, Carlo Colantuoni, Kenneth B Margulies, Thomas P Cappola, Gordon F Tomaselli

**Affiliations:** 1 Department of Medicine, Division of Cardiology, Johns Hopkins University, Baltimore, Maryland, USA; 2 Department of Biostatistics, The Bloomberg School of Public Health, Johns Hopkins University, Baltimore, Maryland, USA; 3 Penn Cardiovascular Institute, University of Pennsylvania School of Medicine, Philadelphia, Pennsylvania, USA

## Abstract

**Background:**

By studying genome-wide expression patterns in healthy and diseased tissues across a wide range of pathophysiological conditions, DNA microarrays have revealed unique insights into complex diseases. However, the high-dimensionality of microarray data makes interpretation of heterogeneous gene expression studies inherently difficult.

**Results:**

Using a large-scale analysis of more than 40 microarray studies encompassing ~2400 mammalian tissue samples, we identified a common theme across heterogeneous microarray studies evident by a robust genome-wide inverse regulation of metabolic and cell signaling pathways: We found that upregulation of cell signaling pathways was invariably accompanied by downregulation of cell metabolic transcriptional activity (and vice versa). Several findings suggest that this characteristic gene expression pattern represents a new principle of mammalian transcriptional regulation. First, this coordinated transcriptional pattern occurred in a wide variety of physiological and pathophysiological conditions and was identified across all 20 human and animal tissue types examined. Second, the differences in metabolic gene expression predicted the magnitude of differences for signaling and all other pathways, i.e. tissue samples with similar expression levels of metabolic transcripts did not show any differences in gene expression for all other pathways. Third, this transcriptional pattern predicted a profound effect on the proteome, evident by differences in structure, stability and post-translational modifications of proteins belonging to signaling and metabolic pathways, respectively.

**Conclusions:**

Our data suggest that in a wide range of physiological and pathophysiological conditions, gene expression changes exhibit a recurring pattern along a transcriptional axis, characterized by an inverse regulation of major metabolic and cell signaling pathways. Given its widespread occurrence and its predicted effects on protein structure, protein stability and post-translational modifications, we propose a new principle for transcriptional regulation in mammalian biology.

## Background

Transcriptional profiling by DNA microarrays allows the simultaneous quantitative analysis of tens of thousands of transcripts in a single experiment. By applying transcriptional profiling technology to healthy and diseased tissues across a wide range of pathophysiological conditions, DNA microarrays have revealed unique insights into complex disease patterns. However, the high-dimensionality of microarray data makes interpretation of heterogeneous gene expression studies inherently difficult. One of the main challenges in the analysis of microarray data is to identify common underlying biological themes by integrating multiple similar experiments. A frequent approach to this problem is to extract common genes from these gene lists and then subject these genes to enrichment analysis by grouping them into pathways.

In a previous study examining failing and non-diseased dog hearts, we observed an intriguing reciprocal transcriptional regulation of selected cell signaling and metabolic processes [1]. To extend this initial observation beyond myocardial tissue and selected pathways, we used a systems biology approach based on KEGG pathways (**K**yoto **E**ncyclopedia of **G**enes and **G**enomes [2]) in a large collection of ~2400 mammalian tissue samples derived from more than 20 diseased and non-diseased tissues. As a result, we identified a robust genome-wide reciprocal regulation of metabolic and cell signaling pathways which was present across all 20 different tissues examined.

## Results

**Figure 1:**
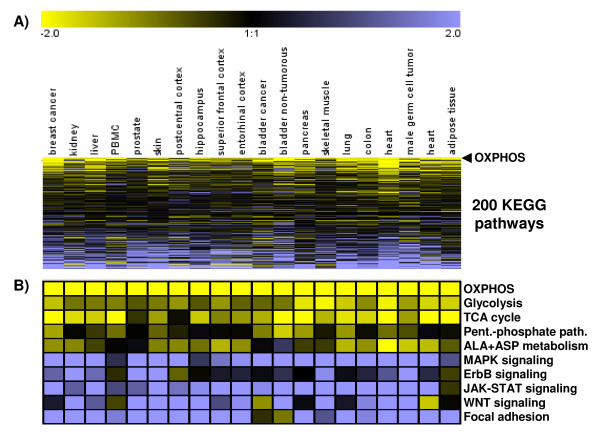
**(A) and (B). Inverse regulation of major metabolic and cell signaling KEGG pathways**. For 20 different human tissues, KEGG pathways were compared between the ten samples displaying the highest and the lowest values of OXPHOS gene expression (study and sample characteristics are listed in Additional Files 2 and 3). The directional regulation of 200 major KEGG pathways (number of up- *minus *down-regulated genes in a given KEGG pathway normalized to the total number of regulated genes within a study) was color-coded with yellow and blue representing low and high expression of the pathways, respectively. KEGG pathways were then sorted according to their similarity to "oxidative phosphorylation" which is represented by the first row (labeled OXPHOS). Metabolic pathways were consistently positively correlated with each other and negatively correlated with the expression of cell signaling pathways. ALA+ASP metabolism = alanine and aspartate metabolism.

**Figure 2:**
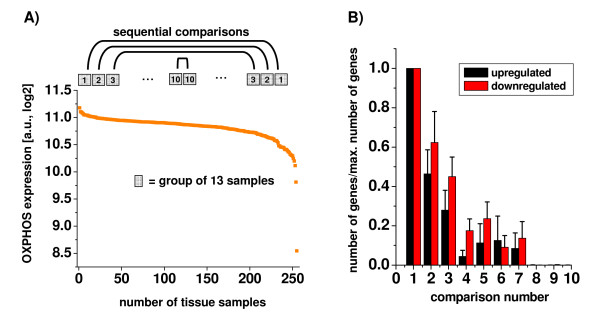
**(A) and (B). Sequential comparisons of tissue samples with highest vs. lowest OXPHOS expression**. In five large microarray datasets (≥ 180 samples each; GSE5406 myocardium, GSE10780 breast tissue, GSE11223 colon, GSE11375 blood mononuclear cells, GEO13507 bladder tissue, Additional File 2), samples were first ranked according to the average expression of all genes belonging to the KEGG pathways of oxidative phosphorylation (OXPHOS). Then, gene expression was compared between group of samples containing between 10 and 13 tissue samples (depending on the size of the dataset) with the highest and lowest OXPHOS expression, the second-highest and second-lowest OXPHOS expression, and so on, using Significance Analysis of Microarrays (SAM) with a false discovery rate (FDR) of 5%. The largest differences were observed for the samples with the highest and lowest OXPHOS gene expression (comparison "1"). For the remaining comparisons (numbered "2"-"10"), the number of differentially expressed transcripts declined rapidly and was zero for samples showing only minor differences in OXPHOS expression levels (comparisons "8"-"10"). Panel B shows the mean ± SEM of all five datasets.

We examined gene expression patterns across 20 large microarray datasets of different human tissues by comparing, in each tissue type, the 10 samples with the highest vs. the lowest gene expression of transcripts belonging to the KEGG pathway of oxidative phosphorylation (OXPHOS) using Significance Analysis of Microarrays [3]. The differentially expressed genes were then grouped into KEGG pathways and depicted as a heat map where KEGG pathways were sorted based on their similarity to OXPHOS expression. A highly coordinated transcriptional response pattern became apparent, as all major metabolic pathways were positively correlated to OXPHOS expression, while cell signaling pathways were inversely correlated to OXPHOS (Figures 1A, B, and Additional Files 1A-1C; detailed study and sample characteristics are listed in Additional Files 2 and 3). What is more, using serial comparisons of large microarray datasets of human colon, myocardial, bladder, leukocytes and breast cancer samples, we found that the total number of differentially expressed genes declined monotonically when tissue samples with decreasing differences in OXPHOS expression were compared to each other (Figures 2A and 2B). Finally, tissue samples with similar expression levels of metabolic transcripts did not show any differences in gene expression (Figure 2B, comparisons 8-10), that is, the differences in metabolic gene expression predict the magnitude of differences for signaling and all other pathways. Thus, the highly coordinated genome-wide transcriptional response which was observed in gene expression datasets of both malignant and non-malignant tissue impacts on the pattern (Figures 1A and 1B) and magnitude (Figure 2B) of the observed gene expression changes.

**Figure 3:**
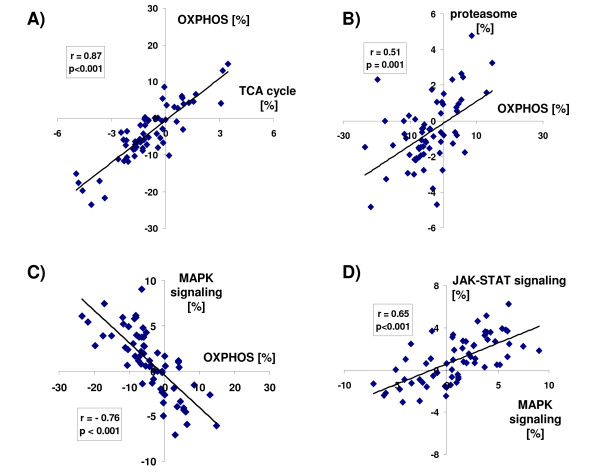
**Correlation of KEGG pathway gene expression**. There is a positive correlation between OXPHOS and TCA ('citrate') cycle **(A) **and between the KEGG pathways of OXPHOS and proteasome **(B)**. OXPHOS and MAPK signaling pathways are negatively correlated **(C)**, while signaling pathways (e.g. JAK-STAT and MAPK) are positively correlated to each other **(D)**. Plots represent net direction of regulation of a KEGG pathway, i.e. number of up- *minus *down-regulated genes in relation to the total number of regulated genes within a study. Correlation plots include all 64 animal and human myocardial microarray studies listed in Additional File 2.

**Figure 4:**
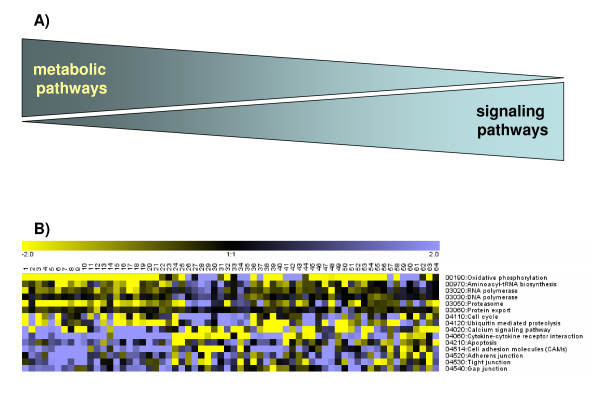
**Patterns of pathway regulation**. **(A) **A schematic of reciprocal correlation of metabolic and signaling pathways in mammalian transcription. **(B) **Relation of major KEGG pathways to OXPHOS. The directional regulation of 14 major KEGG pathways (number of up- *minus *down-regulated genes in a given KEGG pathway normalized to the total number of regulated genes within a study) was color-coded with yellow and blue representing low and high expression of the pathways, respectively. The cellular pathways of "protein export", "cell cycle" and ubiquitin-mediated proteolysis" were positively correlated with OXPHOS, while "calcium-mediated signaling", and structural components important for cell-cell contact (e.g. "cell adhesion molecules", "tight junctions", "gap junctions", "adherens junctions") were negatively correlated with OXPHOS.

To test the hypothesis that the majority of gene expression changes invariably occur along the metabolic - signal transduction axis, we examined gene expression patterns of diverse pathophysiological processes, such as malignant growth, heart failure of ischemic and non-ischemic origin, atrial fibrillation, ageing, liver cirrhosis, psoriasis, diabetes, malaria and inflammatory bowel disease (a complete list of the datasets is given in Additional File 2). When the net direction of regulation between the MAPK and OXPHOS pathways was compared across *all *human and animal microarray studies, defined as the number of up- *minus *down-regulated genes of these KEGG pathways expressed as percentage of the total number of regulated genes within a study, a negative correlation was found (Figure 3C), whereas TCA-cycle and OXPHOS pathways as well as JAK-STAT and MAPK pathways showed a positive correlation (Figure 3A and 3D, respectively). Remarkably, the tight regulation extended beyond KEGG pathways important for metabolic and signaling functions, as evident by the positive correlation between OXPHOS and proteasomal transcripts (Figure 3B), as well as KEGG pathways of "protein export", "cell cycle" and ubiquitin-mediated proteolysis" (Figure 4B). In contrast, "calcium-mediated signaling", and structural components important for cell-cell contact (e.g. "cell adhesion molecules", "tight junctions", "gap junctions", "adherens junctions") were negatively correlated with OXPHOS (Figure 4B; the complete list is given in Additional Files 1A-1C;). Taken together, these data suggest that in a wide range of physiological and pathophysiological conditions, gene expression changes are not random, but instead exhibit a recurring pattern along a transcriptional axis which is characterized by an inverse regulation of major metabolic and cell signaling pathways (Figure 4A). Importantly, transcriptional changes along this axis accounted for >80% of the transcriptional alterations across all datasets (as defined by the number of KEGG pathways that show a statistically significant Pearson correlation coefficient to the OXPHOS pathway, p < 0.05).

**Figure 5:**
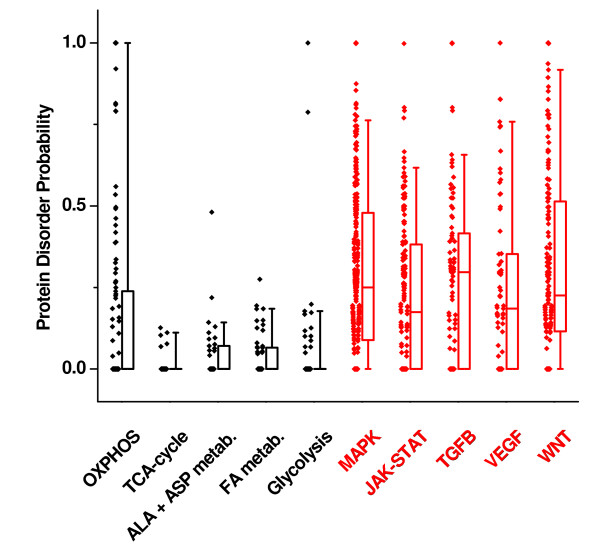
**Disorder Probability of Proteins of KEGG pathways**. KEGG pathways for metabolic genes were more likely to consist of proteins with a higher degree of order, whereas signal transduction pathways include proteins with a higher degree of disorder (p < 0.01, Wilcoxon-test). For each KEGG pathway, boxplots delineate the median value as well as the 25^th ^and 75^th ^percentiles. Raw data, i.e. score representing protein disorder probability (0 and 1 representing low and high degree of disorder, respectively) are plotted as diamonds next to the boxplots. ALA+ASP metabolism = alanine and aspartate metabolism.

**Figure 6:**
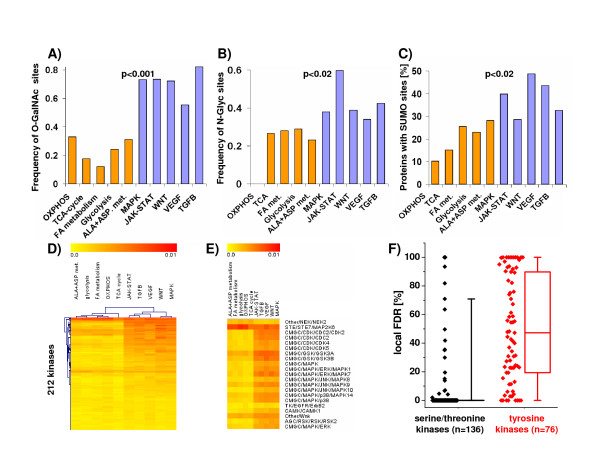
**Post-translational modifications in 5 metabolic vs. 5 signaling pathways**. **(A) **Predicted mucin-type O-glycosylation (O-GalNAc), **(B) **N-glycosylation and **(C) **SUMOylation (Small Ubiquitin-like Modifier) sites are more frequent in signaling (blue bars) vs. metabolic pathways (orange bars). (D)-(F) Predicted frequency of 212 kinase phosphorylation sites (normalized to number and length of proteins within a given pathway to enable comparison across groups). Panel D represents a hierarchical clustering using all 212 kinases (Euclidean distance); the predicted frequency of a given kinase phosphorylation site is color-coded with yellow and red representing low and high expression, respectively. Panel E highlights 21 kinases from Panel D. Only two kinase phosphorylation sites were found to be enriched in metabolic pathways (NEK2 and MAPK2K6). (F) Signaling pathways showed a statistically significant overrepresentation of serine/threonine phosphorylation sites, and to a lesser degree tyrosine phosphorylation sites compared to metabolic pathways (p < 0.01, Wilcoxon-test). For each KEGG pathway, boxplots delineate the median value as well as the 25^th ^and 75^th ^percentiles. The raw data, i.e. individual values for local FDR for the comparison of 5 metabolic vs. 5 signaling pathways are plotted as diamonds next to the boxplots.

The significance of this transcriptional pattern is highlighted by its predicted impact on the proteome: First, significant differences in protein structure were noted between proteins of metabolic vs. signaling pathways. Intrinsically unstructured proteins (IUPs) lack a rigid 3D structure and possess an increased exposed surface area, facilitating interaction with multiple targets [4,5]. These and other properties are ideal for proteins that mediate signaling, transcription and coordinate regulatory events, where binding to multiple partners in high-specificity/low-affinity interactions are paramount [5]. In line with this finding, intrinsic disorder is found in disproportionately higher frequency in proteins belonging to cell signaling compared with metabolic pathways (Figure 5). Second, posttranslational modifications such as phosphorylation can affect the abundance or half-life of certain IUPs [6,7]. Computational studies using phosphorylation site-prediction methods have suggested that unstructured regions are enriched for sites that can be post-translationally modified [8]. We analyzed the predicted occurrence of mucin-type O-glycosylation (O-GalNAc), N-glycosylation, SUMOylation (**S**mall **U**biquitin-like **M**odifier) and 212 kinase phosphorylation sites and found that these post-translational modification sites were significantly enriched in signaling compared to metabolic pathways (Figures 6A-F). Of note, differences in tyrosine phosphorylation sites between metabolic and signaling pathways were not as pronounced as differences in serine/threonine phosphorylation sites, with the latter being significantly enriched in signaling pathways (Figure 6F). Overall, this indicates that proteins of the signaling pathways are not only the source but also a preferred target of post-translational modification, which may be an important mechanism for fine-tuning their function and possibly also controlling their availability.

## Discussion

Cells react to changes in their environment by a coordinated transcriptional response. Using a meta-analysis of more than 40 diverse microarray studies which included different microarray platforms (long and short oligonucleotide arrays, cDNA and bead microarrays) and different methods of normalizations (MAS5, RMA, GC-RMA, VSN, LOWESS), we demonstrate a robust interaction between gene expression in signaling and metabolic pathways. While metabolic pathways were positively correlated to each other, they were negatively correlated to signal transduction pathways. Several findings suggest that this characteristic gene expression pattern represents a novel paradigm for mammalian transcriptional regulation. First, this coordinated transcriptional pattern occurred in a wide variety of physiological and pathophysiological conditions and was identified in all 20 different tissue types examined. Importantly, it occurred independently of the proliferative potential of the underlying tissue, as the inverse regulation of metabolism and signal transduction was observed in terminally differentiated organs like brain and heart, but also in more rapidly dividing malignant tumors. Second, and most strikingly, these changes in steady-state mRNA levels predict a profound effect on the proteome, as KEGG cell signaling pathways are characterized by an increased magnitude of IUPs as compared to metabolic and biosynthetic pathways. The lack of a rigid 3D structure in IUPs is thought to provide several functional advantages, including conformational flexibility to interact with multiple targets, increased interaction surface area, and accessible post-translational modification sites [4,5]. These and other properties are ideal for proteins that mediate signaling, transcription and coordinate regulatory events, where binding to multiple partners and high-specificity/low-affinity interactions play a crucial role [5]. The critical role of IUPs in signaling is further supported by the finding that eukaryotic proteomes, characterized by their rich interaction networks, are highly enriched in IUPs compared to prokaryotes [9]. An increase of IUPs has been associated with perturbed cellular signaling in a wide range of pathological conditions such as cancer, diabetes, and neurodegenerative diseases; thus, intracellular levels of IUPs need to be tightly controlled [10]. Gsponer et al. demonstrated that IUPs as a class had a significantly shorter half-life and lower abundance compared to highly structured proteins in both unicellular and multicellular organisms, suggesting an evolutionarily conserved pattern [10]. Consistent with its role as an ATP-consuming proteolytic system [11], gene expression of proteasomal degradation pathways was positively correlated with metabolic pathways (Figures 3B and 4B). In addition to D- and KEN-boxes, ubiquitin proteasome-dependent degradation is mediated by the N-end-rule and PEST-mediated degradation pathways. Consistent with the shorter protein half-life of IUPs compared to structured proteins [10], recent studies have found IUPs to contain a significantly greater fraction of PEST motifs (regions rich in proline, glutamic acid, serine, and threonine), while no differences were noted for the N-end-rule pathway [10,12]. Importantly, the 20S proteasome can distinguish between intrinsically unstructured and other proteins, as it can digest IUPs under conditions in which native, and even molten globule states, are resistant to degradation [13]. In line with this finding, it has been suggested that the 20S proteasome degradation assay provides a powerful system for operational definition of IUPs [13]. While protein degradation is not determined by a single characteristic, but is a multi-factorial process that shows large protein-to-protein variations [14], it is tempting to speculate that an increased abundance of proteins belonging to metabolic pathways contributes to the down-regulation of signaling pathways via concurrent up-regulation of proteasomal degradation pathways.

## Conclusions

In summary, proteins in signaling and metabolic pathways have fundamentally different properties ranging from inversely regulated transcriptional patterns (Figures 1 and 3), abundance and stability of respective mRNAs to underlying differences in the translational rate, protein abundance and stability [10]. Additionally, profound differences in post-translational modifications exist between signaling and metabolic pathways, as evident by differences in SUMOylation, mucin-type O-glycosylation, N-glycosylation and serine/threonine phosphorylation sites (Figure 6). Ultimately, this novel transcriptional pattern provides a unifying concept for the interpretation of heterogeneous and multi-dimensional microarray datasets, as the dynamic interaction between cellular signaling and metabolic pathways impacts on the quantity (Figure 2B) and pattern (Figures 1, 3 and 4) of the observed gene expression changes. Given the widespread occurrence of this transcriptional pattern and the predicted differences in IUPs, protein stability and post-translational modifications, we propose the reciprocal relationship between metabolic and signaling pathways as a new canonical principle for transcriptional regulation in mammalian biology.

### Study Limitations

In the present study, we noted a striking and robust reciprocal correlation of transcriptional changes between metabolic and signaling pathways. Importantly, correlations do not prove cause and effect. Therefore, we can not determine whether transcriptional changes in metabolic activity anticipate changes in signaling pathways or vice versa. While this study was centered on pathway analysis, future studies will need to identify individual genes or hub nodes that connect metabolic and signaling pathways. In addition, the role of up- and down-stream regulatory events, e.g. transcription factors, miRNAs, splicing, 3' end termination and/or stability of mRNAs need to be examined.

Future studies will need to address the role of this transcriptional pattern in various disease processes. While the association of IUPs with various disease processes might suggest that down-regulation of metabolism and up-regulation of signaling pathways is a common theme in a wide range of disease processes, we found this generalization is not universal. This could be related to a different baseline level of OXPHOS activity in various tissues and cancer specimens and/or differences in tissue handling. Clearly, future studies need to address whether this transcriptional pattern will help in refining the distinction between diseased and non-diseased tissue samples.

## Methods

### Gene Expression Data

Public datasets were obtained from the GEO database [15]. A detailed summary of all datasets used in the present meta-analysis is given in Additional File 2. The criteria for the selection of the dataset were as follows: (1) whole-genome coverage of microarray platforms (covering ≥ 20,000 transcripts; the only exception was the comparison between human adult and fetal hearts, for which whole-genome microarray datasets were not publicly available), (2) quality of normalization procedure: comparable levels of mean signal intensity and variance of signal intensity across experimental groups, (3) non-myocardial tissue datasets had to include at least 50 samples and (4) human myocardial datasets had to have more than ten non-failing samples.

### Statistical Analysis

To determine differentially expressed genes, unpaired two-class Significance Analysis of Microarrays (SAM) was used [3]. Differences in gene expression were regarded as statistically significant if a false discovery rate (FDR) of q<0.05 was achieved. Functional annotation of differentially expressed genes was based on the KEGG pathways database. Overrepresentation of specific KEGG pathways in a gene set was statistically analyzed by the Database for Annotation, Visualization and Integrated Discovery (DAVID) [16]. The net regulation of a pathway was defined as number of up- *minus *down-regulated transcripts of a KEGG pathway expressed as percentage of the total number of regulated genes within a study. Clustering of the expression of KEGG pathways and phosphorylation sites was done using Genesis [17].

Batch prediction of long disordered regions was carried out using the IUPforest-L software, based on the Moreau-Broto autocorrelation function of amino acid indices (AAIs) and other physicochemical features of the primary sequences [18]. Non-parametrical rank tests (Kolmogorov-Smirnoff and Wilcoxon) incorporated into StatView (SAS Institute Inc., NC, USA) were used to determine statistical significance for the distribution of IUP across metabolic and signaling pathways. Batch prediction of N-glycosylation, mucin-type O-glycosylation, SUMOylation and protein kinase phosphorylation sites were carried out using NetNGlyc 1.0 http://www.cbs.dtu.dk/services/NetNGlyc, NetOGlyc 3.1 [19], SUMOsp 2.0 [20], and GPS 2.1 [21], respectively.

## Abbreviations

IUP: Intrinsically Unstructured Proteins; KEGG: Kyoto Encyclopedia of Genes and Genomes; OXPHOS: Oxidative Phosphorylation; SAM: Significance Analysis of Microarrays; DAVID: Database for Annotation, Visualization and Integrated Discovery; GEO: Gene Expression Omnibus.

## Authors' contributions

ASB conceived the study, carried out the experiments and drafted the manuscript. AK and CC provided assistance with the bioinformatic and statistical analysis, respectively; KBM and TPC participated in study design. GFT conceived the study and drafted the manuscript. All authors read and approved the final manuscript.

## Supplementary Material

Additional file 1 **Graphical representation of 200 KEGG pathways sorted based on their similarity to OXPHOS expression**. For 20 different human tissues, KEGG pathways were compared between the ten samples displaying the highest and the lowest values of OXPHOS gene expression (each study-ID with sample characteristics are listed in the tables in Additional Files 2 and 3). The directional regulation of 200 major KEGG pathways (number of up- *minus *down-regulated genes in a given KEGG pathway normalized to the total number of regulated genes within a study) was color-coded with yellow and blue representing low and high expression of the pathways, respectively. KEGG pathways were then sorted according to their similarity to "oxidative phosphorylation" which is represented by the top row in Additional File 1A. Metabolic pathways were consistently positively correlated with each other and negatively correlated with the expression of cell signaling pathways. Click here for file

Additional file 2 **List of gene expression datasets used in the present study**. The study-ID, tissue type, Gene Expression Omnibus (GEO) accession number, species, sample characteristics, comparison, microarray type and methods of normalization are given for each dataset. Click here for file

Additional file 3 **List of human tissues samples with high vs. low OXPHOS gene activity**. The tissue type, study-ID (Gene Expression Omnibus (GEO) accession number), sample-ID and clinical characteristics are given for samples with high and low OXPHOS gene activity. Click here for file
