# Supplementary material for: Reciprocal regulation of metabolic and signaling pathways
Source: BMC Genomics. 2010 Mar 24;11:197. doi: 10.1186/1471-2164-11-197 (PMC2861677; doi:10.1186/1471-2164-11-197)

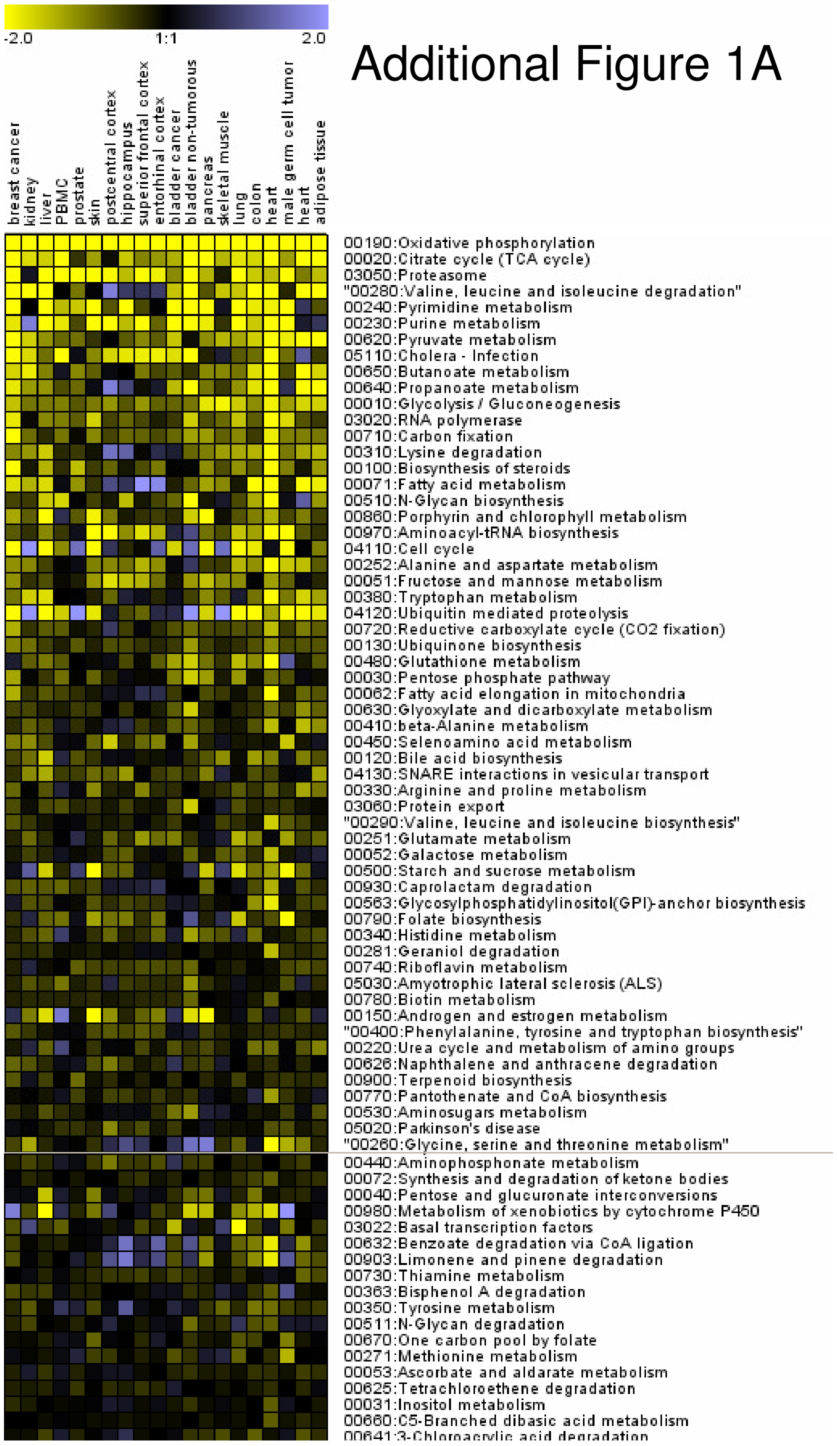

Additional Figure 1A

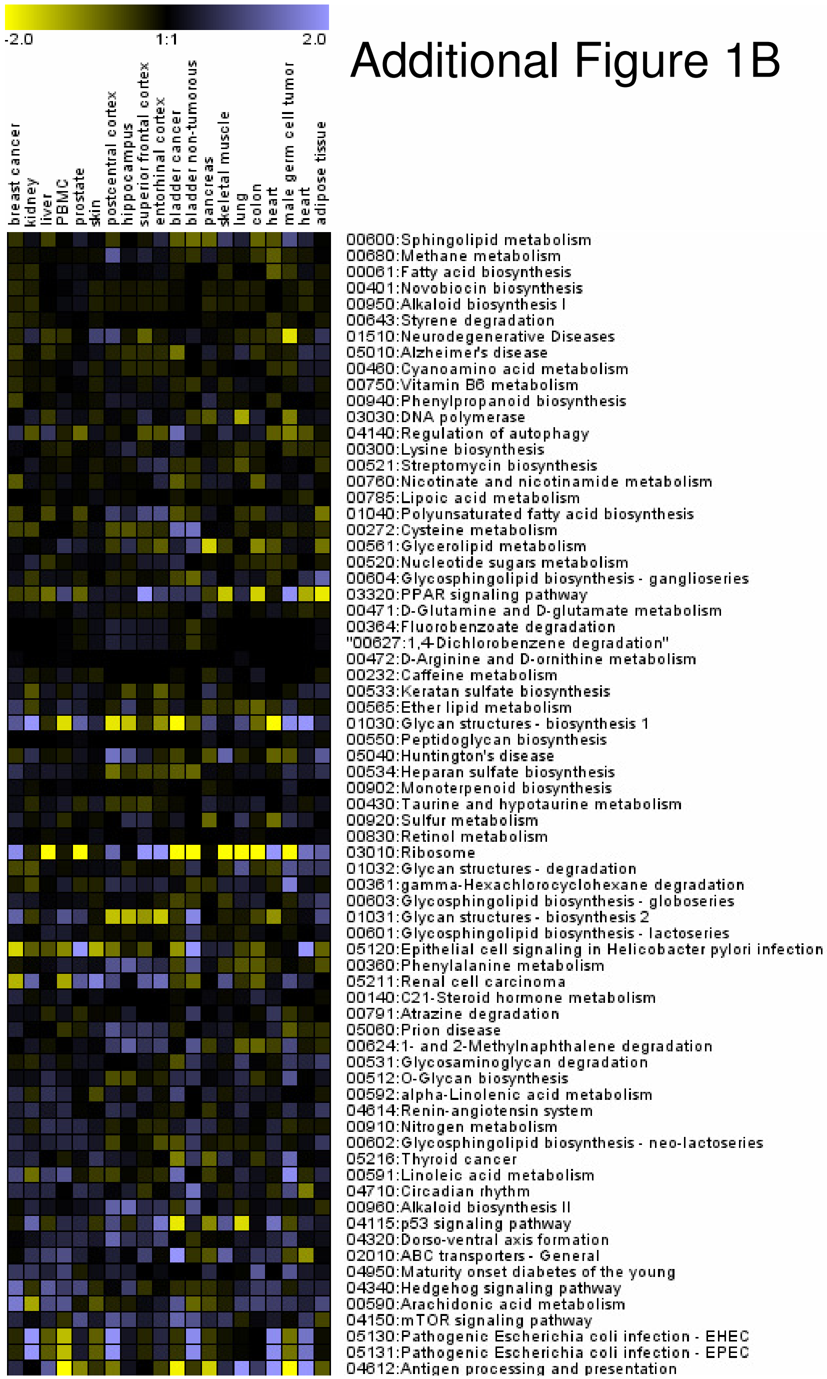

Additional Figure 1B

Additional Figure 1C

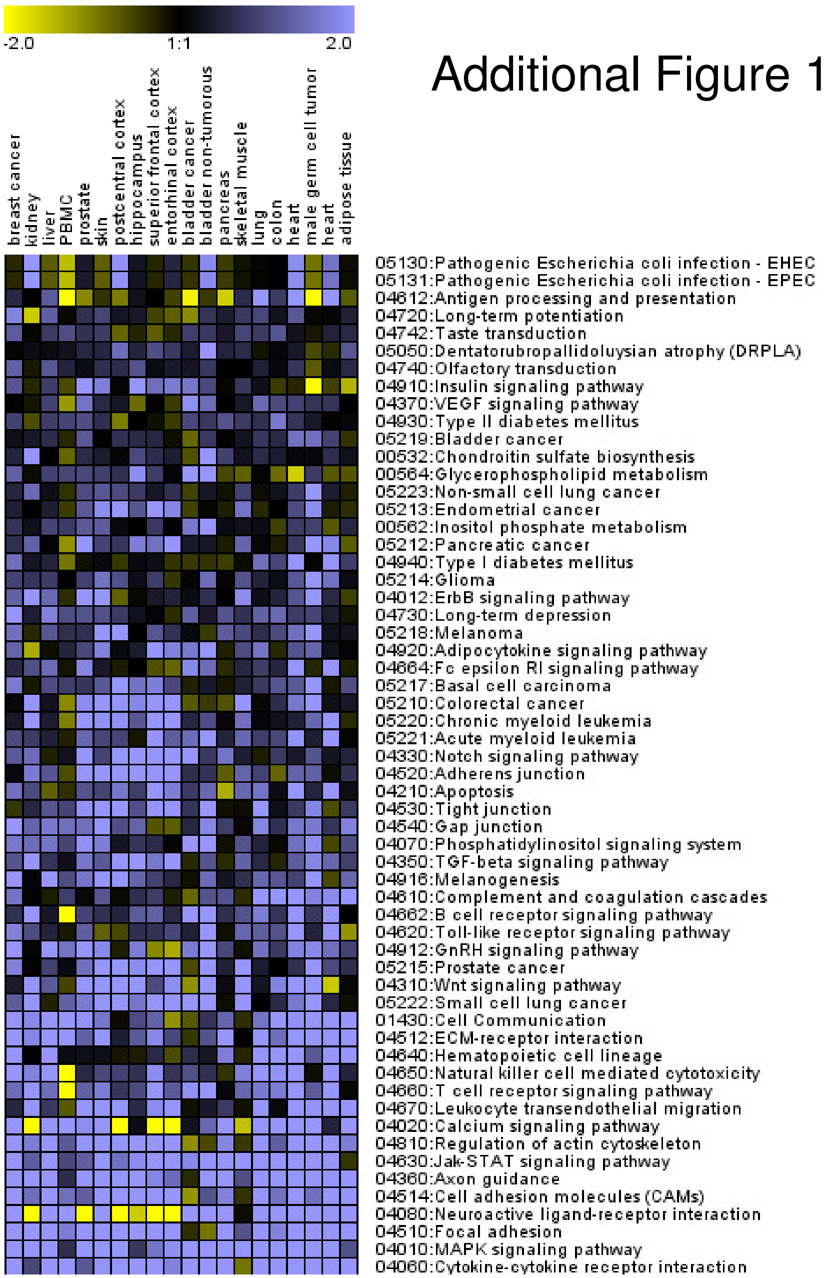

Supplement: Additional file 1 — Graphical representation of 200 KEGG pathways sorted based on their similarity to OXPHOS expression. For 20 different human tissues, KEGG pathways were compared between the ten samples displaying the highest and the lowest values of OXPHOS gene expression (each study-ID with sample characteristics are listed in the tables in Additional Files 2 and 3). The directional regulation of 200 major KEGG pathways (number of up- minus down-regulated genes in a given KEGG pathway normalized to the total number of regulated genes within a study) was color-coded with yellow and blue representing low and high expression of the pathways, respectively. KEGG pathways were then sorted according to their similarity to "oxidative phosphorylation" which is represented by the top row in Additional File 1A. Metabolic pathways were consistently positively correlated with each other and negatively correlated with the expression of cell signaling pathways. [file 1471-2164-11-197-S1.PDF]
