# Supplementary material for: Reciprocal regulation of metabolic and signaling pathways
Source: BMC Genomics. 2010 Mar 24;11:197. doi: 10.1186/1471-2164-11-197 (PMC2861677; doi:10.1186/1471-2164-11-197)
Supplement: Additional file 2 — List of gene expression datasets used in the present study. The study-ID, tissue type, Gene Expression Omnibus (GEO) accession number, species, sample characteristics, comparison, microarray type and methods of normalization are given for each dataset. [file 1471-2164-11-197-S2.PDF]

Additional File 2. Description of gene expression datasets (page 1/2)

manuscript ID	tissue type	GEO accession	Species	sample characteristics	comparison	microarray	normalization
1	breast	GSE5364	human	control (n=13), breast cancer (n=183)	OXPHOS high (n=10) vs. OXPHOS low (n=10)	Affymetrix	MAS5.0
2	kidney	GSE11151	human	control (n=5), various renal cancer (n=62)	OXPHOS high (n=10) vs. OXPHOS low (n=10)	Affymetrix	MAS5.0
3	liver	GSE14323	human	control (n=19), liver cancer ± cirrhosis (n=96)	OXPHOS high (n=10) vs. OXPHOS low (n=10)	Affymetrix	RMA
4	PBMCs	GSE5418	human	control (n=22), malaria (n=49)	OXPHOS high (n=10) vs. OXPHOS low (n=10)	Affymetrix	MAS5.0
5	prostate	GSE6956	human	control (n=20), prostate cancer (n=69)	OXPHOS high (n=10) vs. OXPHOS low (n=10)	Affymetrix	MAS5.0
6	skin	GSE14905	human	control (n=21), psoriasis (n=61)	OXPHOS high (n=10) vs. OXPHOS low (n=10)	Affymetrix	RMA
7	postcentral cortex	GSE11882	human	n=43, various age groups, non-diseased	OXPHOS high (n=10) vs. OXPHOS low (n=10)	Affymetrix	GC-RMA
8	hippocampus	GSE11882	human	n=43, various age groups, non-diseased	OXPHOS high (n=10) vs. OXPHOS low (n=10)	Affymetrix	GC-RMA
9	superior frontal cortex	GSE11882	human	n=48, various age groups, non-diseased	OXPHOS high (n=10) vs. OXPHOS low (n=10)	Affymetrix	GC-RMA
10	entorhinal cortex	GSE11882	human	n=39, various age groups, non-diseased	OXPHOS high (n=10) vs. OXPHOS low (n=10)	Affymetrix	GC-RMA
11	bladder cancer	GSE13507	human	non-tumorous bladder (n=68)	OXPHOS high (n=10) vs. OXPHOS low (n=10)	Illumina	quantile
12	bladder non-tumorous	GSE13507	human	bladder cancer (n=187)	OXPHOS high (n=10) vs. OXPHOS low (n=10)	Illumina	quantile
13	pancreas	GSE15471	human	control (n=39), pancreatic cancer (n=39)	OXPHOS high (n=10) vs. OXPHOS low (n=10)	Affymetrix	RMA
14	skeletal muscle	GSE5086	human	n=81, various age groups, non-diseased	OXPHOS high (n=10) vs. OXPHOS low (n=10)	Affymetrix	Dchip
15	lung	GSE11969	human	control (n=5), various lung cancers (n=158)	OXPHOS high (n=10) vs. OXPHOS low (n=10)	Agilent	LOWESS
16	colon	GSE11223	human	control (n=73), colitis ulcerosa (n=129)	OXPHOS high (n=10) vs. OXPHOS low (n=10)	Agilent	LOWESS
17	thyroid	GSE5364	human	control (n=16), thyroid cancer (n=35)	OXPHOS high (n=10) vs. OXPHOS low (n=10)	Affymetrix	MAS5.0
18	male germ cell tumor	GSE10783	human	various male germ cell tumors (n=35)	OXPHOS high (n=10) vs. OXPHOS low (n=10)	Affymetrix	RMA
19	adipose tissue	GSE13506	human	subcutaneous adipose tissue samples (n=50)	OXPHOS high (n=10) vs. OXPHOS low (n=10)	Affymetrix	RMA
20	heart	GSE5406	human	non-failing (n=16), ICM+NICM (n=194)	OXPHOS high (n=10) vs. OXPHOS low (n=10)	Affymetrix	RMA
21	heart	GSE1145	human	non-failing (n=14), ICM+NICM (n=80)	OXPHOS high (n=10) vs. OXPHOS low (n=10)	Affymetrix	RMA
22	liver	GSE14323	human	control (n=19), liver cancer ± cirrhosis (n=96)	control (n=19) vs. liver cancer (38)	Affymetrix	RMA
23	liver	GSE14323	human	control (n=19), liver cancer ± cirrhosis (n=96)	control (n=19) vs. cirrhosis (58)	Affymetrix	RMA
24	PBMCs	GSE5788	human	normal CD3+ PBMCs (n=8), T-cell malignancy (n=6)	control CD3+ PBMCs (n=8) vs. T-cell malignancy (n=6)	Affymetrix	MAS5.0
25	liposarcoma	GSE12972	human	liposarcoma (n=19), liposarcoma with doxorubicin (n=19)	liposarcoma (n=19) vs. liposarcoma with doxorubicin (n=19)	Affymetrix	MAS5.0
26	breast	GSE5364	human	control (n=13), breast cancer (n=183)	control (n=13) vs. breast cancer (n=183)	Affymetrix	MAS5.0
27	liver	GSE5364	human	normal liver (n=8), liver tumor (n=9)	normal liver (n=8) vs. liver tumor (n=9)	Affymetrix	MAS5.0
28	lung	GSE5364	human	normal lung (n=9), lung tumor (n=15)	control lung (n=9) vs. lung tumor (n=15)	Affymetrix	MAS5.0
29	skin	GSE14905	human	control (n=21), psoriasis (n=61)	control skin (n=21) vs. psoriasis (n=61)	Affymetrix	RMA
30	kidney	GSE11151	human	control (n=5), various renal cancer (n=62)	renal carcinoma (n=26) vs. papillary carcinoma (n=19)	Affymetrix	MAS5.0
31	bladder cancer	GSE13507	human	non-tumorous bladder (n=68), bladder cancer (n=187)	non-tumorous bladder (n=68) vs. bladder cancer (n=187)	Illumina	quantile
32	prostate	GSE6956	human	control (n=20), prostate cancer (n=69)	control prostate (n=20) vs. prostate cancer (n=69)	Affymetrix	MAS5.0
33	pancreas	GSE15471	human	control (n=39), pancreatic cancer (n=39)	control pancreas (n=39) vs. pancreatic cancer (n=39)	Affymetrix	RMA

OXPHOS = oxidative phosphorylation, RMA= robust multi-array average

Additional File 2. Description of gene expression datasets (page 2/2)

manuscript ID	tissue type	GEO accession	Species	sample characteristics	comparison	microarray	normalization
34	lung	GSE11969	human	control (n=5), various lung cancers (n=158)	control lung (n=5) vs. various lung cancers (n=158)	Agilent	LOWESS
35	thyroid	GSE5364	human	control (n=16), thyroid cancer (n=35)	control thyroid (n=16) vs. thyroid cancer (n=35)	Affymetrix	MAS5.0
36	colon	GSE11223	human	control (n=73), colitis ulcerosa (n=129)	control colon (n=73) vs. colitis ulcerosa (n=129)	Agilent	LOWESS
37	PBMCs	GSE5418	human	control (n=22), malaria (n=49)	control PBMCs (n=22) vs. experimental malaria (n=22)	Affymetrix	MAS5.0
38	postcentral cortex	GSE11882	human	n=43, various age groups, non-diseased	young, age<52yrs. (n=19) vs. old, age>64yrs. (n=24)	Affymetrix	GC-RMA
39	hippocampus	GSE11882	human	n=43, various age groups, non-diseased	young, age<52yrs. (n=18) vs. old, age>64yrs. (n=25)	Affymetrix	GC-RMA
40	superior frontal cortex	GSE11882	human	n=48, various age groups, non-diseased	young, age<52yrs. (n=22) vs. old, age>64yrs. (n=26)	Affymetrix	GC-RMA
41	heart	GSE1145	human	non-failing (n=14), ICM+NICM (n=80)	non-failing (n=14) vs. ICM+NICM (n=80)	Affymetrix	RMA
42	heart	GSE3586	human	non-failing (n=15), NICM (n=13)	non-failing (n=15) vs. NICM (n=13)	cDNA Unigene 37K	VSN
43	heart	GSE5406	human	non-failing (n=16), ICM+NICM (n=194)	non-failing (n=16) vs. ICM+NICM (n=194)	Affymetrix	RMA
44	heart	GSE9800	human	non-failing (n=11), ICM+NICM (n=19)	non-failing (n=11) vs. ICM+NICM (n=19)	Agilent	Lowess
45	heart	GSE3530	mouse	wild-type (n=3), MKK7D TG (n=6)	wild-type (n=3) vs. MKK7D transgenic (n=6)	Affymetrix	MAS5.0
46	heart	GSE3530	mouse	wild-type (n=6), MKK3bE TG (n=5)	wild-type (n=6) vs. MKK3bE transgenic (n=5)	Affymetrix	MAS5.0
47	heart	GSE8000	mouse	wild-type (n=8), LMNA TG (n=6)	wild-type (n=8) vs. LMNA TG (n=6)	Affymetrix	MAS5.0
48	heart	GSE12413	mouse	FVB-non-failing (n=10), FVB-ISO-heart failure (n=12)	FVB-non-failing (n=10) vs. FVB-ISO-heart failure (n=12)	Affymetrix	MAS5.0
49	heart	GSE6970	mouse	non-failing (n=8), transverse aortic constriction (n=8)	non-failing (n=8) vs. transverse aortic constriction (n=8)	Affymetrix	MAS5.0
50	heart	GSE5247	dog	non-failing (n=4), heart failure (n=8)	non-failing (n=4) vs. heart failure (n=8)	Affymetrix	MAS5.0
51	heart	GSE9794	dog	non-failing (n=3), heart failure (n=12)	non-failing (n=3) vs. heart failure (n=12)	Affymetrix	MAS5.0
52	heart	GSE11015	dog	non-failing (n=3), heart failure (n=3)	non-failing (n=3) vs. heart failure (n=3)	Affymetrix	MAS5.0
53	heart	GSE14327	dog	non-failing (n=11), heart failure (n=10)	non-failing (n=11) vs. heart failure (n=10)	Agilent	quantile
54	heart	GSE20665	dog	non-failing (n=11), heart failure (n=7), atrial tissue	non-failing (n=11) vs. heart failure (n=7)	Agilent	quantile
55	heart	GSE4963	rat	non-failing (n=4), heart failure (n=10)	non-failing (n=4) vs. heart failure (n=10)	Operon	Lowess
56	heart	GSE4963	rat	non-failing (n=4), heart failure+DITPA (n=6)	non-failing (n=4) vs. heart failure+DITPA (n=6)	Operon	Lowess
57	heart	GSE2240	human	sinus rhythm (n=20), permanent atrial fibrillation (n=10)	sinus rhythm (n=20) vs. permanent atrial fibrillation (n=10)	Affymetrix	RMA
58	heart	GSE2051, 2061, 2062	human	adult (n=7), fetal (n=11)	adult (n=7) vs. fetal (n=11)	Agilent	Lowess
59	heart	GSE2240	human	ventricle (n = 5), atria (n=30)	ventricle (n = 5) vs. atria (n=30)	Affymetrix	RMA
60	heart	GSE14327, GSE20665	dog	ventricle (n = 58), atria (n=18)	ventricle (n = 58) vs. atria (n=18)	Agilent	quantile
61	heart	mousedevelopment.org	mouse	ventricle (n = 3), atria (n=3)	ventricle (n = 3) vs. atria (n=3)	cDNA	dye-bias
62	heart	GSE5085	rat	sedentary (n=4), physical exercise (n=8)	physical exercise (n=8) vs. sedentary rats (n=4)	Affymetrix	RMA
63	heart	GSE6880	rat	control (n=3), diabetic (n=3)	control (n=3) vs. diabetic (n=3)	Affymetrix	MAS5.0
64	heart	GSE5606	rat	control (n=7), diabetic (n=7)	control (n=7) vs. diabetic (n=7)	Affymetrix	MAS5.0

ICM = ischemic cardiomyopathy, NICM = non-ischemic cardiomyopathy, HF= heart failure, FVB = mouse strain, MKK7D and MKK3bE are MAPK genes, LMNA = lamin A, DITPA = Thyroid Hormone Analog
RMA = robust multi-array average, VSN = variance-stabilizing normalization.
