# Supplementary material for: Reciprocal regulation of metabolic and signaling pathways
Source: BMC Genomics. 2010 Mar 24;11:197. doi: 10.1186/1471-2164-11-197 (PMC2861677; doi:10.1186/1471-2164-11-197)
Supplement: Additional file 3 — List of human tissues samples with high vs. low OXPHOS gene activity. The tissue type, study-ID (Gene Expression Omnibus (GEO) accession number), sample-ID and clinical characteristics are given for samples with high and low OXPHOS gene activity. [file 1471-2164-11-197-S3.PDF]

Additional Table 3. Human tissues samples with high vs. low OXPHOS gene activity.

Tissue	Study-ID	OXPHOS high		.	OXPHOS low		.
		Sample-ID	Clinical characteristics	.	Sample-ID	Clinical characteristics	.
breast	GSE5364	GSM121784	Breast tumor sample 111	.	GSM121713	Breast tumor sample 41	.
		GSM121766	Breast tumor sample 94	.	GSM121716	Breast tumor sample 44	.
		GSM121698	Breast tumor sample 26	.	GSM121865	Breast Normal sample 9	.
		GSM121750	Breast tumor sample 78	.	GSM121740	Breast tumor sample 68	.
		GSM121741	Breast tumor sample 69	.	GSM121862	Breast Normal sample 6	.
		GSM121797	Breast tumor sample 124	.	GSM121860	Breast Normal sample 4	.
		GSM121793	Breast tumor sample 120	.	GSM121701	Breast tumor sample 29	.
		GSM121779	Breast tumor sample 106	.	GSM121852	Breast tumor sample 179	.
		GSM121788	Breast tumor sample 115	.	GSM121770	Breast tumor sample 98	.
		GSM121785	Breast tumor sample 112	.	GSM121811	Breast tumor sample 138	.
kidney	GSE11151	GSM281281	chromophobe renal cell cancer; chRCC_H	.	GSM281288	conventional renal cell cancer; cRCCa_HD140	.
		GSM281282	chromophobe renal cell cancer; chRCC_H	.	GSM281340	rhabdoid tumor of kidney; RhT_HD4	.
		GSM281283	chromophobe renal cell cancer; chRCC_H	.	GSM281343	Wilms' tumor; WT_RK34	.
		GSM281284	chromophobe renal cell cancer; chRCC_H	.	GSM281306	conventional renal cell cancer; cRCCb_HD2	.
		GSM281336	renal oncocytoma; RO_HD1175	.	GSM281300	conventional renal cell cancer; cRCCb_HA312	.
		GSM281337	renal oncocytoma; RO_HD171A	.	GSM281294	conventional renal cell cancer; cRCCa_HD34	.
		GSM281338	renal oncocytoma; RO_HD30	.	GSM281302	conventional renal cell cancer; cRCCb_HA463	.
		GSM281339	renal oncocytoma; RO_HD37	.	GSM281287	conventional renal cell cancer; cRCCa_HD114	.
		GSM281319	papillary renal cell cancer; pRCCa_HD108	.	GSM281310	conventional renal cell cancer; cRCCb_HD93	.
		GSM281307	conventional renal cell cancer; cRCCb_HD	.	GSM281299	conventional renal cell cancer; cRCCb_HA306	.
liver	GSE14323	GSM358143	liver tissue with HCC	.	GSM358125	liver tissue with HCC	.
		GSM358130	liver tissue with HCC	.	GSM358129	liver tissue with HCC	.
		GSM358131	liver tissue with HCC	.	GSM358158	liver tissue with HCC	.
		GSM358133	liver tissue with HCC	.	GSM358127	liver tissue with HCC	.
		GSM358172	liver tissue with cirrhosis	.	GSM358200	liver tissue with cirrhosisHCC	.
		GSM358142	liver tissue with cirrhosis	.	GSM358135	liver tissue with HCC	.
		GSM358152	liver tissue with HCC	.	GSM358206	liver tissue with cirrhosis	.
		GSM358149	liver tissue with cirrhosis	.	GSM358196	liver tissue with cirrhosisHCC	.
		GSM358207	liver tissue with HCC	.	GSM358222	Normal liver tissue	.
		GSM358141	liver tissue with cirrhosis	.	GSM358173	liver tissue with HCC	.

Additional Table 3. Human tissues samples with high vs. low OXPHOS gene activity.

Tissue	Study-ID	OXPHOS high		OXPHOS low	
		Sample-ID	Clinical characteristics	Sample-ID	Clinical characteristics
PBMC	GSE5418	GSM123739	treated malaria-Cameroon	GSM123769	baseline uninfected
		GSM123737	acute malaria-Cameroon	GSM123756	baseline uninfected
		GSM123781	acute malaria-Cameroon	GSM123784	baseline uninfected
		GSM123772	acute malaria-Cameroon	GSM123761	baseline uninfected
		GSM123766	acute malaria-Cameroon	GSM123734	experimental malaria-infected
		GSM123741	acute malaria-Cameroon	GSM123777	experimental malaria-infected
		GSM123757	experimental malaria-infected	GSM123740	baseline uninfected
		GSM123749	treated malaria-Cameroon	GSM123738	experimental malaria-infected
		GSM123743	treated malaria-Cameroon	GSM123763	baseline uninfected
		GSM123753	acute malaria-Cameroon	GSM123762	experimental malaria-infected
prostate	GSE6956	GSM160428	Surrounding normal prostate tissue patien	GSM160378	Prostate tumor patient 37
		GSM160420	Surrounding normal prostate tissue protat	GSM160382	Prostate tumor patient 41
		GSM160430	Surrounding normal prostate tissue D	GSM160377	Prostate tumor patient 36
		GSM160431	Surrounding normal prostate tissue patien	GSM160351	Prostate tumor patient 9
		GSM160422	Surrounding normal prostate tissue protat	GSM160391	Prostate tumor patient 51
		GSM160429	Surrounding normal prostate tissue patien	GSM160387	Prostate tumor patient 46
		GSM160419	Pooled non-tumor tissue 2	GSM160365	Prostate tumor patient 24
		GSM160424	Surrounding normal prostate tissue patien	GSM160386	Prostate tumor patient 45
		GSM160409	Surrounding normal prostate tissue patien	GSM160414	Prostate tumor patient 74
		GSM160425	Surrounding normal prostate tissue patien	GSM160396	Prostate tumor patient 56
skin	GSE14905	GSM372360	Lesion Skin, LS-28	GSM372286	Normal Skin, Normal-1
		GSM372362	Lesion Skin, LS-29	GSM372319	Uninvolved Skin, NS-7
		GSM372334	Lesion Skin, LS-15	GSM372287	Normal Skin, Normal-2
		GSM372346	Lesion Skin, LS-21	GSM372297	Normal Skin, Normal-12
		GSM372322	Lesion Skin, LS-8	GSM372305	Normal Skin, Normal-20
		GSM372338	Lesion Skin, LS-17	GSM372321	Uninvolved Skin, NS-8
		GSM372340	Lesion Skin, LS-18	GSM372294	Normal Skin, Normal-9
		GSM372320	Lesion Skin, LS-7	GSM372296	Normal Skin, Normal-11
		GSM372367	Lesion Skin, LS-33	GSM372288	Normal Skin, Normal-3
		GSM372327	Lesion Skin, LS-11	GSM372288	Normal Skin, Normal-3

Additional Table 3. Human tissues samples with high vs. low OXPHOS gene activity.

Tissue	Study-ID	OXPHOS high		OXPHOS low	
		Sample-ID	Clinical characteristics	Sample-ID	Clinical characteristics
postcentral cortex	GSE11882	GSM300202	PostcentralGyrus_female_91yrs_indiv27	GSM300310	PostcentralGyrus_male_22yrs_indiv85
		GSM300226	PostcentralGyrus_female_90yrs_indiv40	GSM300277	PostcentralGyrus_male_20yrs_indiv77
		GSM300283	PostcentralGyrus_female_36yrs_indiv79	GSM300322	PostcentralGyrus_female_47yrs_indiv88
		GSM300256	PostcentralGyrus_male_69yrs_indiv67	GSM300281	PostcentralGyrus_male_20yrs_indiv78
		GSM300326	PostcentralGyrus_male_69yrs_indiv93	GSM300175	PostcentralGyrus_male_45yrs_indiv12
		GSM300302	PostcentralGyrus_male_20yrs_indiv83	GSM300314	PostcentralGyrus_male_42yrs_indiv86
		GSM300232	PostcentralGyrus_female_37yrs_indiv45	GSM300306	PostcentralGyrus_male_33yrs_indiv84
		GSM300273	PostcentralGyrus_female_26yrs_indiv76	GSM300318	PostcentralGyrus_male_45yrs_indiv87
		GSM300166	PostcentralGyrus_female_91yrs_indiv10	GSM300291	PostcentralGyrus_female_44yrs_indiv80
		GSM300216	PostcentralGyrus_male_97yrs_indiv34	GSM300259	PostcentralGyrus_male_40yrs_indiv68
hippocampus	GSE11882	GSM300294	Hippocampus_female_48yrs_indiv81	GSM300178	Hippocampus_male_95yrs_indiv14
		GSM300301	Hippocampus_male_20yrs_indiv83	GSM300329	Hippocampus_female_91yrs_indiv94
		GSM300280	Hippocampus_male_20yrs_indiv78	GSM300215	Hippocampus_male_97yrs_indiv34
		GSM300298	Hippocampus_female_30yrs_indiv82	GSM300187	Hippocampus_female_45yrs_indiv17
		GSM300276	Hippocampus_male_20yrs_indiv77	GSM300190	Hippocampus_female_74yrs_indiv18
		GSM300219	Hippocampus_female_34yrs_indiv35	GSM300193	Hippocampus_female_99yrs_indiv2
		GSM300185	Hippocampus_male_91yrs_indiv16-07	GSM300325	Hippocampus_male_69yrs_indiv93
		GSM300317	Hippocampus_male_45yrs_indiv87	GSM300272	Hippocampus_female_26yrs_indiv76
		GSM300197	Hippocampus_female_74yrs_indiv21	GSM300333	Hippocampus_male_75yrs_indiv96
		GSM300174	Hippocampus_male_45yrs_indiv12	GSM300255	Hippocampus_male_69yrs_indiv67
superior frontal cortex	GSE11882	GSM300254	brain, superior frontal gyrus, male, 21 year	GSM300327	brain, superior frontal gyrus, male, 69 years
		GSM300278	brain, superior frontal gyrus, male, 20 year	GSM318840	brain, superior frontal gyrus, male, 66 years
		GSM300296	brain, superior frontal gyrus, female, 48 ye	GSM300229	brain, superior frontal gyrus, female, 44 years
		GSM300260	brain, superior frontal gyrus, male, 40 year	GSM300191	brain, superior frontal gyrus, female, 74 years
		GSM300292	brain, superior frontal gyrus, female, 44 ye	GSM300167	brain, superior frontal gyrus, female, 91 years
		GSM300221	brain, superior frontal gyrus, female, 34 ye	GSM300288	brain, superior frontal gyrus, male, 69 years
		GSM300315	brain, superior frontal gyrus, male, 42 year	GSM300250	brain, superior frontal gyrus, female, 85 years
		GSM300307	brain, superior frontal gyrus, male, 33 year	GSM300251	brain, superior frontal gyrus, female, 91 years
		GSM300282	brain, superior frontal gyrus, male, 20 year	GSM300213	brain, superior frontal gyrus, female, 74 years
		GSM300319	brain, superior frontal gyrus, male, 45 year	GSM300284	brain, superior frontal gyrus, female, 36 years

Additional Table 3. Human tissues samples with high vs. low OXPHOS gene activity.

Tissue	Study-ID	OXPHOS high		OXPHOS low	
		Sample-ID	Clinical characteristics	Sample-ID	Clinical characteristics
entorhinal cortex	GSE11882	GSM300258	EntorhinalCortex_male_40yrs_indiv68	GSM300328	EntorhinalCortex_female_91yrs_indiv94
		GSM300289	EntorhinalCortex_female_44yrs_indiv80	GSM300285	EntorhinalCortex_male_69yrs_indiv8
		GSM300275	EntorhinalCortex_male_20yrs_indiv77	GSM300324	EntorhinalCortex_male_69yrs_indiv93
		GSM300297	EntorhinalCortex_female_30yrs_indiv82	GSM300214	EntorhinalCortex_male_97yrs_indiv34
		GSM300320	EntorhinalCortex_female_47yrs_indiv88	GSM300261	EntorhinalCortex_male_52yrs_indiv71
		GSM300293	EntorhinalCortex_female_48yrs_indiv81	GSM300332	EntorhinalCortex_male_75yrs_indiv96
		GSM300304	EntorhinalCortex_male_33yrs_indiv84	GSM300189	EntorhinalCortex_female_74yrs_indiv18
		GSM300242	EntorhinalCortex_female_64yrs_indiv52	GSM300252	EntorhinalCortex_male_21yrs_indiv66
		GSM300279	EntorhinalCortex_male_20yrs_indiv78	GSM300228	EntorhinalCortex_female_44yrs_indiv42
		GSM300316	EntorhinalCortex_male_45yrs_indiv87	GSM300267	EntorhinalCortex_male_86yrs_indiv73
bladder cancer	GSE13507	GSM340703	Primary bladder cancer BT099	GSM340741	Primary bladder cancer BT138
		GSM340677	Primary bladder cancer BT073	GSM340667	Primary bladder cancer BT063
		GSM340670	Primary bladder cancer BT066	GSM340692	Primary bladder cancer BT088
		GSM340676	Primary bladder cancer BT072	GSM340638	Primary bladder cancer BT034
		GSM340653	Primary bladder cancer BT049	GSM340685	Primary bladder cancer BT081
		GSM340706	Primary bladder cancer BT102	GSM340626	Primary bladder cancer BT022
		GSM340698	Primary bladder cancer BT094	GSM340605	Primary bladder cancer BT001
		GSM340751	Primary bladder cancer BT148	GSM340613	Primary bladder cancer BT009
		GSM340647	Primary bladder cancer BT043	GSM340668	Primary bladder cancer BT064
		GSM340663	Primary bladder cancer BT059	GSM340606	Primary bladder cancer BT002
bladder non-tumorous	GSE13507	GSM340548	Surrounding BS038	GSM340592	Surrounding BS129
		GSM340575	Surrounding BS095	GSM340583	Surrounding BS109
		GSM340574	Surrounding BS093	GSM340584	Surrounding BS111
		GSM340600	Surrounding BS169	GSM340547	Surrounding BS017
		GSM340593	Surrounding BS130	GSM340578	Surrounding BS101
		GSM340539	Control C029	GSM340590	Surrounding BS125
		GSM340568	Surrounding BS084	GSM340544	Control C045
		GSM340545	Control C051	GSM340559	Surrounding BS059
		GSM340565	Surrounding BS080	GSM340553	Surrounding BS045
		GSM340557	Surrounding BS052	GSM340586	Surrounding BS115

Additional Table 3. Human tissues samples with high vs. low OXPHOS gene activity.

Tissue	Study-ID	OXPHOS high		.	OXPHOS low		.
		Sample-ID	Clinical characteristics	.	Sample-ID	Clinical characteristics	.
pancreas	GSE15471	GSM388087	sample: normal	.	GSM388153	sample: tumor	.
		GSM388089	sample: normal	.	GSM388093	sample: normal	.
		GSM388107	sample: normal	.	GSM388109	sample: normal	.
		GSM388088	sample: normal	.	GSM388084	sample: normal	.
		GSM388106	sample: normal	.	GSM388095	sample: normal	.
		GSM388086	sample: normal	.	GSM388123	sample: tumor	.
		GSM388126	sample: tumor	.	GSM388097	sample: normal	.
		GSM388092	sample: normal	.	GSM388101	sample: normal	.
		GSM388138	sample: tumor	.	GSM388122	sample: tumor	.
		GSM388119	sample: tumor	.	GSM388111	sample: normal	.
skeletal muscle	GSE5086	GSM114716	Skeletal muscle taken from other anatomi	.	GSM114662	Skeletal muscle taken from rectus abdominis	.
		GSM114712	Skeletal muscle taken from other anatomi	.	GSM114690	Skeletal muscle taken from rectus abdominis	.
		GSM114709	Skeletal muscle taken from other anatomi	.	GSM114651	Skeletal muscle taken from rectus abdominis	.
		GSM114711	Skeletal muscle taken from other anatomi	.	GSM114689	Skeletal muscle taken from rectus abdominis	.
		GSM114699	Skeletal muscle taken from rectus abdomi	.	GSM114677	Skeletal muscle taken from rectus abdominis	.
		GSM114652	Skeletal muscle taken from rectus abdomi	.	GSM114680	Skeletal muscle taken from rectus abdominis	.
		GSM114647	Skeletal muscle taken from rectus abdomi	.	GSM114676	Skeletal muscle taken from rectus abdominis	.
		GSM114707	Skeletal muscle taken from other anatomi	.	GSM114663	Skeletal muscle taken from rectus abdominis	.
		GSM114650	Skeletal muscle taken from rectus abdomi	.	GSM114654	Skeletal muscle taken from rectus abdominis	.
		GSM114710	Skeletal muscle taken from other anatomi	.	GSM114722	Skeletal muscle taken from other anatomical re	.
lung	GSE11969	GSM303153	Patient 09 with small cell lung cancer	.	GSM303028	Patient 033 with adenocarcinoma	.
		GSM303139	Patient 030 with squamous cell carcinoma	.	GSM303034	Patient 039 with adenocarcinoma	.
		GSM303122	Patient 013 with squamous cell carcinoma	.	GSM303003	Patient 008 with adenocarcinoma	.
		GSM303093	Patient 004 with large-cell carcinoma	.	GSM303155	Mixture of normal tissue from 5 different patient	.
		GSM303106	Patient 017 with large-cell carcinoma	.	GSM303157	Mixture of normal tissue from 5 different patient	.
		GSM303109	Patient 002 with large cell neuroendocrine	.	GSM303076	Patient 081 with adenocarcinoma	.
		GSM303123	Patient 014 with squamous cell carcinoma	.	GSM303009	Patient 014 with adenocarcinoma	.
		GSM303144	Patient 035 with squamous cell carcinoma	.	GSM303011	Patient 016 with adenocarcinoma	.
		GSM303121	Patient 012 with squamous cell carcinoma	.	GSM302999	Patient 004 with adenocarcinoma	.
		GSM303128	Patient 019 with squamous cell carcinoma	.	GSM303156	Mixture of normal tissue from 5 different patient	.

Additional Table 3. Human tissues samples with high vs. low OXPHOS gene activity.

Tissue	Study-ID	OXPHOS high			OXPHOS low		
		Sample-ID	Clinical characteristics	.	Sample-ID	Clinical characteristics	.
colon	GSE11223	GSM282898	11383 Normal Uninflamed ascending colo	.	GSM283016	11432 UC Uninflamed sigmoid colon	.
		GSM282974	5272 UC Uninflamed sigmoid colon	.	GSM283041	12479 UC Uninflamed sigmoid colon	.
		GSM282960	5258 UC Uninflamed terminal ileum	.	GSM282908	11393 Normal Uninflamed ascending colon	.
		GSM282976	5274 UC Inflamed descending colon	.	GSM282997	11409 UC Inflamed ascending colon	.
		GSM282912	11397 Normal Uninflamed descending col	.	GSM283050	12488 UC Inflamed sigmoid colon	.
		GSM282923	12458 Normal Uninflamed ascending colo	.	GSM282996	11408 UC Inflamed sigmoid colon	.
		GSM282911	11396 Normal Uninflamed ascending colo	.	GSM282937	5223 UC Inflamed sigmoid colon	.
		GSM282962	5260 UC Uninflamed descending colon	.	GSM282934	5218 UC Inflamed descending colon	.
		GSM282977	5275 UC Uninflamed descending colon	.	GSM283014	11430 UC Inflamed sigmoid colon	.
		GSM283034	12472 UC Uninflamed ascending colon	.	GSM283029	12467 UC Inflamed sigmoid colon	.
thyroid	GSE5364	GSM121985	Thyroid tumor sample 7	.	GSM122027	Thyroid normal sample 16	.
		GSM121998	Thyroid tumor sample 16	.	GSM121993	Thyroid normal sample 2	.
		GSM121989	Thyroid tumor sample 10	.	GSM122020	Thyroid tumor sample 30	.
		GSM121995	Thyroid tumor sample 14	.	GSM122011	Thyroid normal sample 9	.
		GSM122023	Thyroid normal sample 14	.	GSM122013	Thyroid normal sample 10	.
		GSM121981	Thyroid tumor sample 3	.	GSM121994	Thyroid normal sample 3	.
		GSM122006	Thyroid tumor sample 21	.	GSM122029	Thyroid tumor sample 35	.
		GSM122025	Thyroid tumor sample 33	.	GSM122000	Thyroid tumor sample 18	.
		GSM121988	Thyroid tumor sample 9	.	GSM121991	Thyroid tumor sample 12	.
		GSM122017	Thyroid normal sample 11	.	GSM121992	Thyroid tumor sample 13	.
male germ cell tumor	GSE10783	GSM271786	mixed GCT (Teratoma, Choriocarcinoma,	.	GSM271811	pure GCT (Teratoma with secondary somatic m	.
		GSM271789	pure GCT (Embryonal Carcinoma)	.	GSM271793	mixed GCT (Teratoma, Yolk Sac Tumor)	.
		GSM271812	pure GCT (Teratoma with secondary soma	.	GSM271791	mixed GCT (Yolk Sac, Teratoma)	.
		GSM271782	mixed GCT (Synciotrophoblasts, Yolk Sac	.	GSM271794	pure GCT (Teratoma)	.
		GSM271805	mixed GCT (Yolk Sac, Embryonal Carcinc	.	GSM271788	mixed GCT (Yolk Sac, Embryonal Carcinoma, T	.
		GSM271780	mixed GCT (Embryonal Carcinoma, Terat	.	GSM271809	mixed GCT (Yolk Sac, Embryonal Carcinoma, T	.
		GSM271779	GCT (Teratoma with secondary somatic m	.	GSM271798	pure GCT (Yolk Sac Tumor)	.
		GSM271807	mixed GCT (Yolk Sac, Embryonal Carcinc	.	GSM271803	pure GCT (Teratoma)	.
		GSM271799	mixed GCT (Yolk Sac, Embryonal Carcinc	.	GSM271806	pure GCT (Teratoma)	.
		GSM271785	pure GCT (Teratoma)	.	GSM271792	pure GCT (Teratoma with secondary somatic m	.

Additional Table 3. Human tissues samples with high vs. low OXPHOS gene activity.

Tissue	Study-ID	OXPHOS high		.	OXPHOS low		.
		**Sample-ID**	**Clinical characteristics**	.	**Sample-ID**	**Clinical characteristics**	.
heart	GSE5406	GSM123663	human failing LV myocardium, Idiopathic1	.	GSM123639	human nonfailing LV myocardium, NonFailing11	.
		GSM123583	human failing LV myocardium, Ischemic72	.	GSM123529	human failing LV myocardium, Ischemic90	.
		GSM123669	human failing LV myocardium, Ischemic25	.	GSM123712	human failing LV myocardium, Ischemic38	.
		GSM123678	human failing LV myocardium, Ischemic19	.	GSM123703	human failing LV myocardium, Ischemic86	.
		GSM123580	human failing LV myocardium, Ischemic74	.	GSM123532	human failing LV myocardium, Ischemic88	.
		GSM123576	human failing LV myocardium, Ischemic77	.	GSM123649	human nonfailing LV myocardium, NonFailing2	.
		GSM123653	human failing LV myocardium, Idiopathic2	.	GSM123713	human failing LV myocardium, Ischemic35	.
		GSM123680	human failing LV myocardium, Ischemic17	.	GSM123707	human failing LV myocardium, Ischemic41	.
		GSM123544	human failing LV myocardium, Ischemic82	.	GSM123534	human failing LV myocardium, Ischemic85	.
		GSM123577	human failing LV myocardium, Ischemic76	.	GSM123642	human failing LV myocardium, Ischemic37	.
adipose tissue	GSE13506	GSM340514	Subcutaneous adipose tissue, SAT27	.	GSM340535	Subcutaneous adipose tissue, SAT48	.
		GSM340504	Subcutaneous adipose tissue, SAT17	.	GSM340511	Subcutaneous adipose tissue, SAT24	.
		GSM340516	Subcutaneous adipose tissue, SAT29	.	GSM340536	Subcutaneous adipose tissue, SAT49	.
		GSM340491	Subcutaneous adipose tissue, SAT4	.	GSM340498	Subcutaneous adipose tissue, SAT11	.
		GSM340519	Subcutaneous adipose tissue, SAT32	.	GSM340518	Subcutaneous adipose tissue, SAT31	.
		GSM340493	Subcutaneous adipose tissue, SAT6	.	GSM340534	Subcutaneous adipose tissue, SAT47	.
		GSM340501	Subcutaneous adipose tissue, SAT14	.	GSM340499	Subcutaneous adipose tissue, SAT12	.
		GSM340496	Subcutaneous adipose tissue, SAT9	.	GSM340529	Subcutaneous adipose tissue, SAT42	.
		GSM340523	Subcutaneous adipose tissue, SAT36	.	GSM340527	Subcutaneous adipose tissue, SAT40	.
		GSM340515	Subcutaneous adipose tissue, SAT28	.	GSM340517	Subcutaneous adipose tissue, SAT30	.
